# Insights into *TREM2* biology by network analysis of human brain gene expression data

**DOI:** 10.1016/j.neurobiolaging.2013.05.001

**Published:** 2013-12

**Authors:** Paola Forabosco, Adaikalavan Ramasamy, Daniah Trabzuni, Robert Walker, Colin Smith, Jose Bras, Adam P. Levine, John Hardy, Jennifer M. Pocock, Rita Guerreiro, Michael E. Weale, Mina Ryten

**Affiliations:** a Istituto di Genetica delle Popolazioni—CNR, Sassari, Italy; b Reta Lila Weston Institute and Department of Molecular Neuroscience, University College London Institute of Neurology, Queen Square, London, UK; c Department of Medical and Molecular Genetics, King's College London, Guy's Hospital, London, UK; d Department of Genetics, King Faisal Specialist Hospital and Research Centre, Riyadh, Saudi Arabia; e MRC Sudden Death Brain Bank Project, Department of Neuropathology, University of Edinburgh, Edinburgh, UK; f Division of Medicine, University College London, London, UK; g Department of Neuroinflammation, University College London Institute of Neurology, London, UK

**Keywords:** TREM2, Weighted gene co-expression network analysis, Alzheimer's disease, Post-mortem human brain, Microglia, Immune system

## Abstract

Rare variants in *TREM2* cause susceptibility to late-onset Alzheimer's disease. Here we use microarray-based expression data generated from 101 neuropathologically normal individuals and covering 10 brain regions, including the hippocampus, to understand *TREM2* biology in human brain. Using network analysis, we detect a highly preserved *TREM2*-containing module in human brain, show that it relates to microglia, and demonstrate that *TREM2* is a hub gene in 5 brain regions, including the hippocampus, suggesting that it can drive module function. Using enrichment analysis we show significant overrepresentation of genes implicated in the adaptive and innate immune system. Inspection of genes with the highest connectivity to *TREM2* suggests that it plays a key role in mediating changes in the microglial cytoskeleton necessary not only for phagocytosis, but also migration. Most importantly, we show that the *TREM2*-containing module is significantly enriched for genes genetically implicated in Alzheimer's disease, multiple sclerosis, and motor neuron disease, implying that these diseases share common pathways centered on microglia and that among the genes identified are possible new disease-relevant genes.

## 1 Introduction

Alzheimer's disease (AD) is the most important cause of pre-senile dementia worldwide. In the last 3 years, there has been growing evidence for the importance of genetic risk factors in AD pathogenesis with the discovery of 10 new loci from genome-wide association studies (GWAS) (Hollingworth et al., 2011; Naj et al., 2011), and, most recently, the identification of heterozygous rare variants in *TREM2* as a cause of increased susceptibility to late-onset AD (Guerreiro et al., 2012; Jonsson et al., 2012). Given that the *TREM2* variants reported are associated with an odds ratio of 2.90 to 5.05, similar to ApoE4, and given that this is an entirely new AD locus, these findings are of potentially high importance to the understanding of AD pathogenesis (Guerreiro et al., 2012; Jonsson et al., 2012). However, *TREM2* is yet to be extensively studied in this context, and its functions in human brain are not fully understood at present.

*TREM2* encodes a trans-membrane glycoprotein, consisting of an extracellular immunoglobulin-like domain, a transmembrane domain, and a cytoplasmic tail. It is known to be expressed on the cell membrane of a subset of myeloid cells, including dendritic cells, osteoclasts, tissue macrophages, and, most importantly for AD, microglia, the resident macrophages of the central nervous system (Colonna, 2003). Since *TREM2* itself has no intracellular signaling motif, it is thought to be completely dependent on the adaptor protein *TYROBP* (also known as DAP12) for its downstream effects. Consistent with this view is the fact that homozygous loss-of-function mutations in either *TREM2* or *TYROBP* result in Nasu–Hakola disease (Online Mendelian Inheritance in Man number 605086), a rare disease characterized by painful bone cysts, psychotic symptoms, and progressive presenile dementia (Paloneva et al., 2002). Upon *TYROBP* stimulation, an ITAM-src kinase-signaling pathway is initiated leading to cellular activation via a range of downstream cascades (Linnartz et al., 2010) and among other possible functions, phagocytosis. Although the endogenous ligand of *TREM2* remains unknown, *TREM2* has been shown to recognize lipopolysaccharides in the cell wall of bacteria and, on activation, triggers the phagocytic uptake of bacteria and the release of reactive oxygen species (Daws et al., 2003; N'Diaye et al., 2009). Other studies have implicated *TREM2* in the phagocytosis of apoptotic material both in vitro and in experimental autoimmune encephalomyelitis, an animal model for multiple sclerosis. The build-up of apoptotic material and the inability of microglia with reduced *TREM2* expression to clear it may lead to a frustrated pro-inflammatory phenotype, which would explain why microglia with reduced *TREM2* expression, when challenged with apoptotic material also had increased expression of pro-inflammatory cytokines (Stuart et al., 2003).

Despite this information, there is clearly still a great deal that we do not yet know about *TREM2,* and in particular, there is only limited information about its functions in the human brain. Understanding the predominant biological functions of *TREM2* in the brain is clearly of immense importance in the identification of potential therapeutic targets for AD and new candidate genes. In this study, we use a systems-biology approach (Lee et al., 2004; Stuart et al., 2003), based on whole-transcriptome gene expression analysis, to identify co-expressed genes with *TREM2* and to begin to address these questions in human brain tissue. We expression-profiled 788 brain samples sampled from 101 neurologically and neuropathologically normal individuals (10 distinct brain regions each) as part of the UK Human Brain Expression Consortium (UKBEC), and used weighted gene co-expression network analysis (WGCNA) to group genes into modules in an unsupervised manner (Langfelder and Horvath, 2008; Oldham et al., 2006; Oldham et al., 2008; Zhang and Horvath, 2005). A large and growing body of evidence now exists to suggest that this approach is extremely useful in identifying modules of biologically related genes that are not just co-expressed but co-regulated (Konopka et al., 2011; Oldham et al., 2008; Rosen et al., 2011; Winden et al., 2009). In this article, we focus our analysis on the *TREM2*-containing modules and compare through preservation statistics the overlap between *TREM2*-containing modules in different brain regions and different data sets to better understand the function of this gene and its relationship to other known AD-related genes.

## 2 Methods

### 2.1 Human brain samples and analysis of Affymetrix Human Exon 1.0 ST array

Brain samples were collected by the Medical Research Council (MRC) Sudden Death Brain and Tissue Bank (Millar et al., 2007), Edinburgh, UK. All individuals were confirmed to be neuropathologically normal by histology performed on sections prepared from paraffin-embedded brain tissue blocks, and the diagnosis was determined by a consultant neuropathologist. All samples had fully informed consent for retrieval and were authorized for ethically approved scientific investigation (Research Ethics Committee number 10/H0716/3).

Total RNA was isolated from human post-mortem brain tissues using the miRNeasy 96 well kit (Qiagen, Manchester, UK). The quality of total RNA was evaluated by the 2100 Bioanalyzer (Agilent Technologies, Wokingham, UK) and RNA 6000 Nano Kit (Agilent Technologies, Wokingham, UK) before processing with the Ambion WT Expression Kit and Affymetrix GeneChip Whole Transcript Sense Target Labeling Assay, and hybridization to the Affymetrix Exon 1.0 ST Arrays (Affymetrix UK Ltd, High Wycombe, UK) following the manufacturers' protocols. Hybridized arrays were scanned on an Affymetrix GeneChip Scanner 3000 7G (Affymetrix UK Ltd, High Wycombe, UK) and visually inspected for hybridization artefacts. Further details regarding tissue collection, RNA isolation, quality control, and processing have been previously reported (Trabzuni et al., 2011).

All arrays were pre-processed using Robust Multi-array Average (quantile normalization, probe set summary by median polish) with GC background correction (GC-RMA) algorithm (Wu et al., 2004) in Partek Genomics Suite v6.6 (Partek Inc, St. Louis, MO). After re-mapping the Affymetrix probe sets onto human genome build 19 (GRCh37) as documented in the Netaffx annotation file (HuEx-1_0-st-v2 Probeset Annotations, Release 31), we restricted analysis to probe sets that had gene annotation and contained at least 3 probes that hybridized uniquely to a genome position. The resulting expression data were corrected for individual effects (within which are nested post-mortem interval, brain pH, sex, age at death, and cause of death) and experimental batch effects (date of hybridization). Transcript-level expression was calculated for 26,993 genes using Winsorized means (Winsorizing the data below 10% and above 90%). We further restricted to 15,409 transcripts that passed the Detection Above Background (DABG) criteria (*p* < 0.001 in at least 50% of samples in at least 1 brain region), had a coefficient of variation >5%, and expression values of at least 5 in samples in at least 1 brain region.

### 2.2 Analysis of publicly available GEO data sets

Human microarray data sets were downloaded from the Gene Expression Omnibus (GEO; (Edgar et al., 2002)) and ArrayExpress (Parkinson et al., 2011) repositories. The accession numbers of the data sets used were GSE30272 (human brain tissue, Illumina Human 49K Oligo array [HEEBO-7 set], 23), E-MTAB-945 (monocytes, Illumina HumanHT-12 v4.0 expression beadchip (Fairfax et al., 2012), E-TABM-733 (macrophages, Affymetrix GeneChip Human Genome U133 Plus 2.0 (Smith et al., 2009), and GSE34151 (dendritic cells, Illumina HumanHT-12 v4.0 expression beadchip, 26). In the case of all 4 data sets, we restricted our analysis to probes with gene annotation and evidence of detection above background. With regard to the 2 Illumina HumanHT-12 v4.0 data sets, gene annotation was based on the re-mapping of probes as performed by the ReMOAT pipeline (Barbosa-Morais et al., 2010) and probes were required to pass a detection *p* value of <0.01 in at least 50% of samples in a given data set. In the case of the macrophage data set generated using the Affymetrix GeneChip Human Genome U133 Plus 2.0, gene annotation was based on the current Netaffyx annotation file (HG-U133_Plus_2.na33.annot.csv), and probes were required to pass a detection *p*-value of <0.01 in at least 50% of samples with the data set. Finally, in the case of the brain data set (GSE30272), gene annotation was based on the annotation files provided within GEO, and further detection-based probe filtering was performed as described by Colantuoni et al. (Colantuoni et al., 2011). In all cases, the resulting expression data were corrected for experimental batch effects and individual effects (including sex and age when available).

### 2.3 Identification and removal of outlier samples for network analyses

Gene co-expression analyses are particularly sensitive to the presence of outlier samples, and therefore rigorous quality control procedures were used to ensure the highest possible level of quality for each dataset. Outliers were identified by visual inspection after clustering the samples using hierarchical clustering with Euclidean distance as the distance measure. Transcripts clusters showed no relation with other covariates (e.g., age, gender, cause of death, post-mortem interval, pH). We also confirmed that the majority of the identified outliers had low interarray correlation, which is defined as the Pearson correlation coefficient of the expression levels for a given pair of transcripts using all available data available (i.e., <3 standard deviations [SDs] of the average interarray correlation). After outlier removal, we repeated the process to check for additional outliers.

### 2.4 Network construction

We followed a step-by-step network construction and module detection. The network was constructed from the pairwise Pearson correlations between all genes, raising these correlations to a power of 12 while maintaining the information on the correlation signs between genes, thus constructing a “signed” network (Mason et al., 2009). Under this design, only positive correlations among genes are used to construct the network. The selected power used to weight the correlations between genes was chosen to recapitulate scale-free topology (Zhang and Horvath, 2005).

Topological overlap measure (TOM), a pairwise measure of node similarity (i.e., how close the neighbors of a gene are to the neighbors of the other gene) was then calculated. Unlike correlation or adjacency measures, which consider each pair of genes in isolation, TOM considers each pair of genes in relation to all other genes in the network, and it reflects the relative interconnectedness of 2 nodes within their local neighbourhood. The TOM measure allows us to create more robust co-expression relationships (Yip and Horvath, 2007), serving as a filter to exclude spurious or isolated connections during network construction.

Then, by defining a dissimilarity matrix based on TOM (1-TOM), and creating a hierarchical clustering tree based on this dissimilarity matrix, a dynamic tree-cutting algorithm was used to “cut” each dendrogram and define an initial set of modules for each network as branches of the cluster trees (the minimum module size was arbitrarily set to 30 genes). After an initial set of modules was created, correlations among module eigengenes (MEs), defined as the first principal component of the expressions of the genes within the module, are used to merge close modules (we choose a height cut of 0.1 of the ME dendrogram, corresponding to correlation of 0.9, to merge modules). MEs summarize the characteristic expression pattern of a module, and modules with very similar expression profiles will show highly correlated eigengenes. This procedure allows the identification of modules of densely interconnected, coexpressed genes (Oldham et al., 2006; Ravasz and Barabasi, 2003; Zhang and Horvath, 2005), and has been shown to be placing functionally related genes into groups (Eisen et al., 1998).

### 2.5 Preservation analysis

To validate module construction for a reference network, it is desirable to show that modules are reproducible (or preserved) in an independent network. To this end, a simple cross-tabulation analysis of module memberships, and the related Fisher's exact test *p* value can be calculated. In general, to determine whether a module found in a reference data set can also be found in a test data set, module preservation statistics implemented in the WGCNA R software can be used.

For each pair of brain tissues, the overlap between all modules was calculated along with the probability of observing such overlap by chance. The significance of module overlap was assessed using a Fisher's exact test (also known as hypergeometric test) for each of the pairwise overlap. To account for multiple comparisons, a Bonferroni correction was applied on the basis of the number of modules in the comparison networks.

To validate modules consistency across tissues, and to evaluate specifically how well the *TREM2*-containing modules were preserved across tissues, we calculated a composite Z summary statistic that aggregates different module preservation statistics (Langfelder et al., 2011). The composite Z summary statistic can be used to determine whether module nodes remain highly connected in the test network, and to determine whether the connectivity pattern between nodes in the reference network is similar to that in the test network. We used a permutation-based procedure in WGCNA to calculate a Z summary for each module (specifically, randomly permuting the module labels in the test network and calculate corresponding preservation statistics). The higher the preservation Z summary is for a given module in a specific brain tissue, the stronger the evidence that the module is preserved in another brain region. Under the null hypothesis of no module preservation, the preservation Z summary follows an approximately standard normal distribution, and comprehensive simulation studies led to the following thresholds: a module shows no evidence of preservation if its Z summary is less than 2; a Z summary greater than 5 (or 10) indicates moderate (strong) module preservation (Langfelder et al., 2011).

### 2.6 Network connectivity and module memberships

For each network the whole network connectivity (also known as “degree”) for all genes can be calculated, and it describes the relative importance of each gene in the network (Zhang and Horvath, 2005). To identify the most highly connected, or most central genes within each module, referred to as “hubs”, measure of module memberships (MMs) can be used. MMs are derived by first summarizing the expression levels of all genes in each module by the corresponding ME (that is, the first principal component obtained by singular value decomposition). MMs for each gene with respect to each module are defined as the Pearson correlation between the expression level of a given gene and the module ME. This quantity describes the extent to which a gene “belongs” to a module. Values of MM range from −1 (perfect anti-correlation) to 1 (perfect correlation). We used MMs to characterize the importance of *TREM2* within each module for each brain tissue.

### 2.7 Gene set enrichment analysis with DAVID

To evaluate the biological and functional relevance of coexpressed genes within the *TREM2*-containing modules, we used DAVID v6.7 (http://david.abcc.ncifcrf.gov/), the database for annotation, visualization, and integrated discovery (Huang da et al., 2009b). We examined the overrepresentation (i.e., enrichment) of the 3 Gene Ontology (GO) categories (biological processes, cellular components, and molecular function) for each list of coexpressed genes with *TREM2* for each tissue by comparing numbers of significant genes annotated with this biological category with chance.

## 3 Results

### 3.1 Regional distribution of TREM2 mRNA expression in human brain

The human data used here were provided by the UK Human Brain Expression Consortium (Trabzuni et al., 2011) and consisted of 101 control post-mortem brains. All samples originated from individuals with no significant neurological history or neuropathological abnormality, and all were collected by the MRC Edinburgh Brain Bank (Millar et al., 2007) ensuring a consistent dissection protocol and sample handling procedure. A summary of the available demographic details of these samples, including a thorough analysis of their effects on array quality, is provided by Trabzuni et al (Trabzuni et al., 2011). In brief, samples originated from individuals with a male: female ratio of 77:24 (3.2), mean age at death of 51 years (range, 16–83 years), and modal cause of death of ischemic heart disease (59%; full details provided in Supplementary Tables 1 and 2). Up to 10 brain regions were sampled from each individual, resulting in 788 Affymetrix Human Exon 1.0 ST arrays after rigorous removal of outliers. The regions sampled were BA 8 and 9 (frontal cortex [FCTX]), BA17 (occipital cortex [OCTX]), BA 21 and 22 (temporal cortex [TCTX]), intralobular white matter (WHMT), hippocampus (HIPP), thalamus (at the level of the lateral geniculate nucleus (THAL), putamen (at the level of the anterior commissure [PUTM]), substantia nigra (SNIG), the inferior olivary nucleus (sub-dissected from the medulla [MEDU]) and the cerebellar cortex (CRBL).

**Fig. 1:**
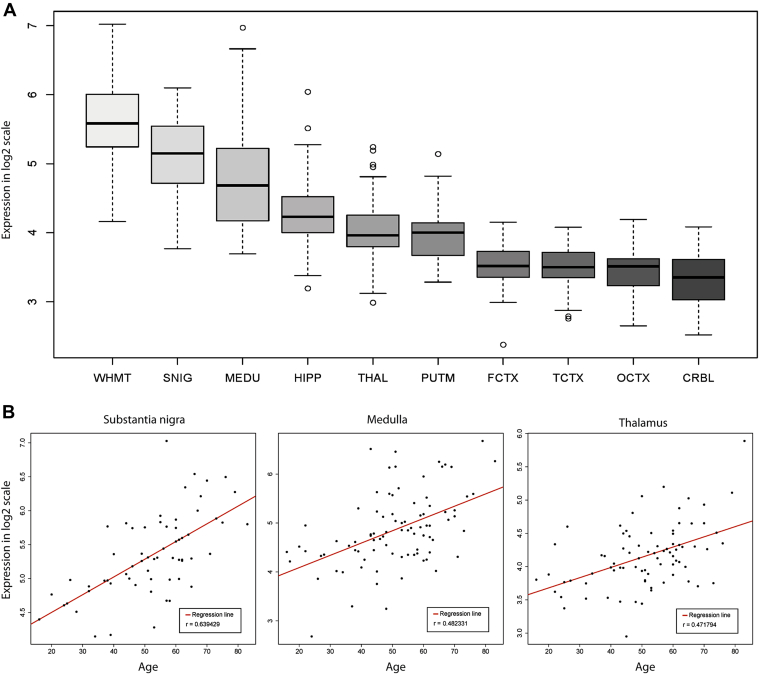
Regional and age-related variability in *TREM2* expression in human brain. (A) Regional distribution of *TREM2* mRNA expression in human brain: box plot of mRNA expression levels for *TREM2* in 10 brain regions, based on microarray experiments and plotted on a log2 scale (*y*-axis). Whiskers extend from the box to 1.5 times the interquartile range. (B) Age-related changes in *TREM2* expression in substantia nigra, medulla, and thalamus, based on microarray experiments and plotted on a log2 scale (*y*-axis).

Fig. 1A shows the regional distribution of mRNA expression for *TREM2* at the gene level for human brains. This demonstrated significant regional differences in *TREM2* gene expression with a 4.96-fold difference (paired *t* test *p* value = 6.63 × 10^−33^) between white matter (the highest-expressing region) and cerebellum (lowest-expressing region). Given the age-dependent and sex-biased incidence of Alzheimer's disease (AD), we investigated *TREM2* expression in each brain region for age- or sex-related changes in expression, while accounting for the potential confounding effects of post-mortem interval, cause of death, brain pH, and experimental batch effects. We found suggestive age-related increases in *TREM2* expression in 3 brain regions: MEDU (*p* = 0.043), THAL (*p* = 0.037), and SNIG (*p* = 0.048, Fig. 1B). Although these findings could not be replicated using the only other publicly available data set covering these brain regions (Kang et al., 2011) (Supplementary Fig. 1), it should be noted that this particular data set contains samples originating from only 18 adults, whereas our study is based on samples originating from 101 individuals. There was no evidence of sex-biased expression of *TREM2* in any brain region.

### 3.2 Weighted Gene Co-expression Network Analysis identifies a TREM2-containing module, which is highly preserved in all brain regions

**Fig. 2:**
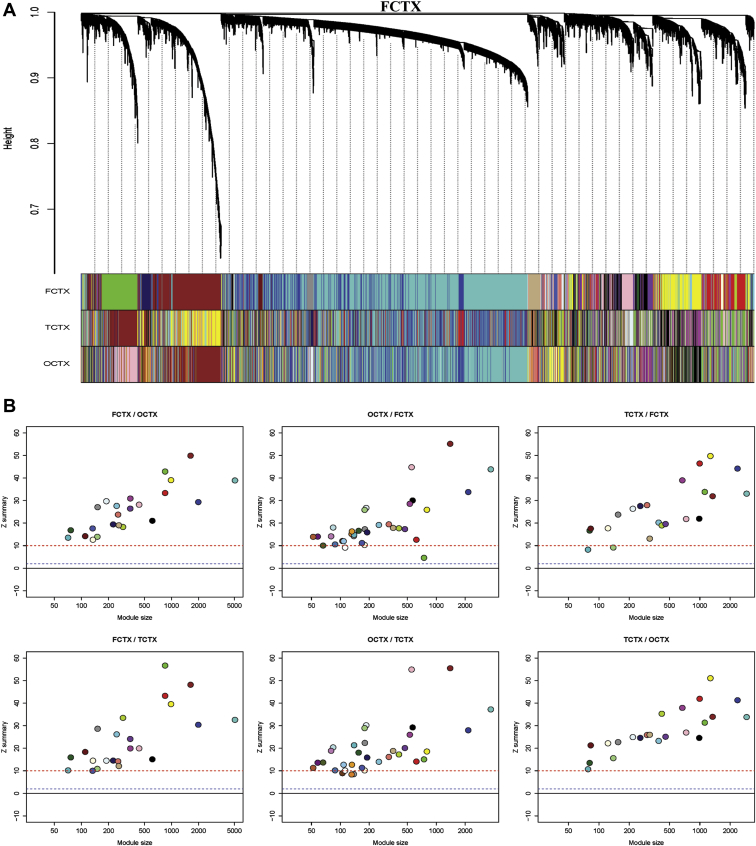
Evidence of high conservation of modules across brain cortical regions. (A) Hierarchical clustering dendrogram for FCTX and equivalent module assignment colours for all 3 cortical regions (frontal cortex [FCTX], temporal cortex [TCTX], occipital cortex [OCTX]). (B) Preservation statistics (Z summary) among the 3 cortical regions, i.e. FCTX/OCTX shows the preservation Z summary statistics for the FCTX modules in OCTX.

To gain insights into the functional organization of the brain transcriptome, we used the WGCNA package (Langfelder and Horvath, 2008; Zhang and Horvath, 2005) and focused on *TREM2*. This analytic approach uses the degree of gene neighbourhood sharing, as defined on the basis of co-expression relationships, to identify groups or modules of genes that are highly co-expressed and (by implication) functionally related (Geschwind and Konopka, 2009; Miller et al., 2008; Oldham et al., 2008; Winden et al., 2009). We used WGCNA to analyze gene expression data on 15,409 transcripts equating to 13,706 genes (passing filtering as described in Methods) to construct signed weighted gene co-expression networks for each of the 10 brain regions (see Methods). On this basis, we identified between 13 and 34 modules per region, depending on the brain region (Table 1). Whole network connectivity for each tissue and *TREM2*-specific connectivity is summarized in the Supplementary Table 3. Before proceeding to investigate *TREM2-*containing modules, we checked the consistency of all the identified modules across the 10 brain regions and particularly within the 3 available neocortical regions (FCTX, TCTX, and OCTX). As would be expected, we observed particularly high module conservation among the 3 cortical regions, as shown in (Fig. 2A and B). These findings are consistent with the study by Oldham et al. (Oldham et al., 2008) that showed similar co-expression preservation in 3 different regions in the brain.

**Fig. 3:**
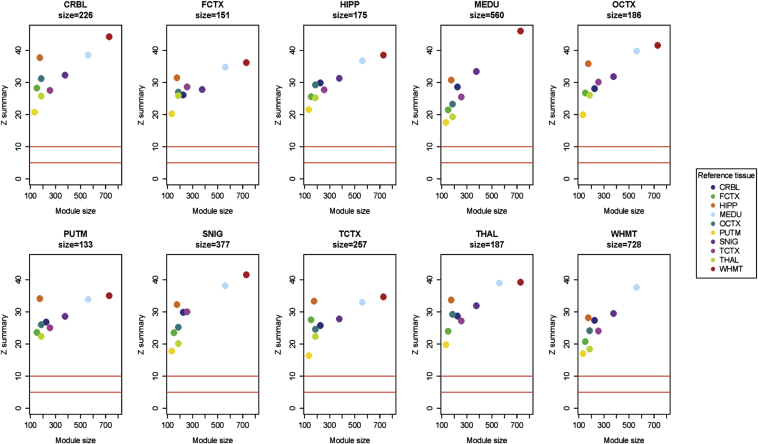
Preservation statistic (Z summary) of each regional *TREM2*-containing module across the equivalent modules in all other human brain regions.

Having established the robustness of the whole network, we checked the preservation statistics for the *TREM2*-containing modules across all 10 brain regions. Table 2 shows the number of shared genes (and percentages) among the *TREM2*-containing modules from each region. We identified 77 genes present in all 10 regional *TREM2*-containing modules. We used Fisher's exact to test for significant overlap in module membership and found that all 45 pairwise comparisons were highly significant (*p* < 1.1 × 10^−3^ = 0.05/45 after Bonferroni correction), indicating a high overlap in module membership among all *TREM2*-containing modules. We also used a permutation test procedure implemented in the WGCNA R package to evaluate preservation across multiple tissues. This procedure produces a summary preservation Z statistic (plotted in Fig. 3) that takes into account connectivity patterns between nodes, not just overlaps in module membership on cross-tabulation (see Methods) (Langfelder et al., 2011). We found that the *TREM2*-containing module is highly conserved in all brain regions.

### 3.3 Independent human brain dataset replicates gene membership found within the TREM2-containing module

For validation purposes, we used an independent and publicly available dataset (Colantuoni et al., 2011), collected from human dorsolateral prefrontal cortex in an extensive series of samples collected from fetal development through aging (GSE30272). We restricted our analysis to adult brain samples (individuals aged 16 years and more, n = 175) to maximize comparability with the UKBEC dataset. Using the same parameters for network construction as were applied to the UKBEC data set, we constructed a signed WGCNA for 15,250 transcripts (representing 10,740 gene symbols). On this basis, we identified 46 modules (size ranging between 37 and 1,969 transcripts; whole network connectivity between 4.6 and 146.0). The *TREM2*-containing module in this dataset contained 255 unique genes symbols (representing 315 transcripts). We observe a highly significant overlap between genes in this module and the genes within the corresponding *TREM2*-containing module in the frontal cortex of the UKBEC dataset. More specifically, we observe 95 overlapping gene symbols (2.2 genes expected by chance, Fisher's exact test *p* value <7 × 10^−142^).

### 3.4 TREM2-containing module is partially preserved in blood-derived macrophages

Given the importance of *TREM2* in the pathogenesis of AD, there is an urgent need to better understand its functions. Although ideally this would be achieved by studying adult human microglia, practically this is challenging. This led us to assess the validity of using other more accessible and potentially relevant human cell types, namely monocytes, macrophages, and dendritic cells. To do this, we used publicly available human whole-genome gene expression data generated on the Illumina HT12 v4 beadchip array and the Affymetrix U133 Plus 2.0 array with large numbers of healthy individuals: (monocytes, E-MTAB-945, N = 188 (Fairfax et al., 2012); macrophages, E-TABM-733, N = 16 (Smith et al., 2009); dendritic cells, GSE34151, N = 69 (Barreiro et al., 2012)). In each case, we started by checking the evidence for *TREM2* expression in these cell types. In keeping with the existing literature (on the basis of microarray data), we were unable to convincingly detect *TREM2* expression in monocytes (percentage of samples passing a detection *p* value of 0.01 = 4.1%), but could detect expression in nonstimulated macrophages and dendritic cells.

We next used WGCNA to assess gene expression data on 11,658 genes in macrophages and 8300 genes in dendritic cells (passing filtering as described in Methods) to construct signed weighted gene co-expression networks (see Methods). Focusing on the *TREM2*-containing module in macrophages and dendritic cells, we found that *TREM2* had high module membership (macrophages and dendritic cells, 61^th^ quantile) but was not a “hub” gene in either cell type. We next assessed preservation of the *TREM2*-containing module across the 2 cell types and in post-mortem human brain tissue. We found that whereas there was no significant preservation of the *TREM2*-containing module between dendritic cells and macrophages (Fisher's exact test *p* value = 0.38) or dendritic cells and brain (Fisher's exact test *p* value = 0.77), there were 13 genes in common between the *TREM2*-containing modules in macrophages and hippocampus (5.62 genes expected by chance, Fisher's exact test *p* = 4.52 × 10^−3^). Although this does suggest significant preservation of the *TREM2*-containing module across these 2 tissue/cell types, it is much weaker than that observed between the 2 brain data sets analyzed (Fisher's exact test *p* value <7 × 10^−142^).

### 3.5 TREM2-containing modules in human brain correspond to a microglial signature

Consistent with the expression of *TREM2* in microglia, the known microglia markers *CX3CR1, ITGAM, AIF1*, *FCER1G,* and *CD68* were present within the *TREM2*-containing module in all brain regions including the independent replication data set. In the case of each of these genes, we measured their module membership (MM), as defined by how well the expression pattern of each gene within the module correlates with the first principal component of gene expression for that module, termed the module eigengene (see Methods). In all cases, the mean MM across all brain regions was above the median (*CX3CR1* 56^th^; *ITGAM* 93^rd^; *AIF1* 82^nd^, *FCER1G* 78^th^, and *CD68* 97^th^ quantile). These findings and, in particular, the high MM of microglia markers, suggest that the *TREM2*-containing modules in human brain strongly relate to the gene expression signature of microglia. It should also be noted that searching for genes related to the major neurotransmitter signaling systems (namely glutamatergic, GABAergic, dopaminergic, cholinergic, serotonergic, histaminergic, glycinergic, purinergic, adenosinergic, noradrenergic, and adrenergic) demonstrated only the presence of the purinergic and adenosinergic receptors (*ADORA3*, *P2RX4*, *P2RY12,* and *P2RY13*) within the *TREM2*-containing modules, suggesting that this module is very unlikely to relate to any neuronal population. Similarly, no oligodendrocyte markers (*OLIGO1, OLIGO2, MAG*, *MOG,* and *MOBP*) were detected and of the astrocyte markers (*GFAP*, *S100B,* and *ALDOC*); only *GFAP* (a marker of activated astrocytes) was present in the *TREM2*-containing module in white matter (quantile = 38^th^).

**Fig. 4:**
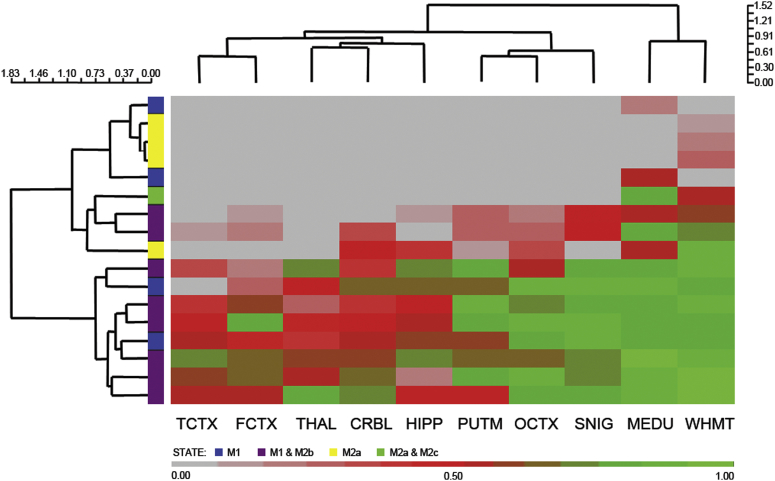
Hierarchical clustering of microglial markers across all 10 brain regions based on module membership. Genetic markers of microglial state that were not detected in a given regional *TREM2*-containing module are depicted in gray (and have a module membership of 0).

We also investigated gene membership within the *TREM2*-containing modules to see if we could infer the predominant microglial activation state relevant to these modules. Despite the distinct origin of microglia (Ginhoux et al., 2010), these cells (like peripheral macrophages) have been classified as M1- or M2-like (with sub-classification of M2a, M2b, and M2c). Whereas the M1 phenotype is adopted in response to activation with lipopolysaccharide or interferon-γ and denotes a pro-inflammatory state, the M2 phenotype is adopted in response to interleukin-4 (Gordon, 2003). We investigated features of 62 genetic markers (as described elsewhere (David and Kroner, 2011); Supplementary Table 4) of the M1 and M2 activation states. Of these, 17 genes were members of at least 1 of the regional *TREM2*-containing modules. These included the following: 5 genes associated with the M2a activation state (*IGF1*, *PDGFB*, *PDGFC*, *TGFB1,* and *MSR1*); 7 genes contributing to the major histocompatibility complex (MHC) class II protein complex which has been associated with both the M1 and M2b activation state (*CD74*, *HLA-DMA*, *HLA-DMB*, *HLA-DPA1*, *HLA-DPB1*, *HLA-DQA2,* and *HLA-DRA*); 4 genes associated with the M1 activation state (*FCGR2A*, *FCGR2B*, *FCGR3B,* and *CXCL10*); and finally *CD86*, which (like MHC class II) has been associated with both the M1 and M2b states. Because we detected markers for both the M1 and M2 activation states, we used MM as a basis for hierarchical clustering of genes to better assess microglia state in different brain regions. These data are presented in Fig. 4 and show that the dominant markers (based on MM) were members of the MHC class II protein complex, and that markers of the M2a complex were detected only in white matter and, to a lesser extent, the medulla.

### 3.6 TREM2 is a “hub” gene within the TREM2-containing module in the hippocampus and white matter among other brain regions

Within each *TREM2*-containing module, WGCNA allows us to identify the most highly connected genes, or central genes, referred to as “hubs,” as defined by a high MM (as described above). The MM for *TREM2* was above the median in all brain regions, including the replication data set (88^th^ quantile within this module). In fact, *TREM2* was in the top 90^th^ quantile in the hippocampus, white matter, medulla, putamen, and substantia nigra (Table 3), indicating that *TREM2* is clearly a hub gene in these 5 tissues.

Based on this definition of “hub,” namely, MM in the top 90^th^ quantile in at least 5 brain regions, we identified a further 15 genes of interest: *APBB1IP*, *C3AR1*, *CYBB*, *DOCK2*, *DOCK8*, *NCKAP1L*, *ADORA3*, *CD68*, *FYB*, *PTPRC*, *TBXAS1*, *ADAM28*, *ITGAM*, *METTL9,* and *CD84*. This would suggest that although 980 genes are identified as members of the *TREM2*-containing module across all 10 brain regions, *TREM2* is actually part of a select group of genes driving module function.

### 3.7 TREM2-containing modules in the human brain are enriched for genes implicated not only in the innate but also the adaptive immune system

Gene Ontology (GO) enrichment analysis was performed on the *TREM2*-containing modules in each brain region using the Database for Annotation, Visualization and Integrated Discovery (DAVID) (Huang da et al., 2009a, 2009b). The results of this analysis are summarized in Table 4 and provided in full in Supplementary Table 5. This demonstrated that the *TREM2*-containing modules from all 10 brain regions were highly enriched for genes related to immune response (minimum Bonferroni-corrected *p* value = 1.65 × 10^−47^). This finding is clearly consistent with the known expression of *TREM2* on microglia (Colonna, 2003). In keeping with the existing literature, GO enrichment analysis provided strong evidence for a role for *TREM2* in the innate immune response (minimum Bonferroni-corrected *p* value = 3.44 × 10^−12^) and, more specifically, lysosome activity in all brain regions. This was demonstrated by the significant enrichment of genes classified by GO to lytic vacuoles and lysosomes in 7 of the 10 regional *TREM2*-containing modules including the hippocampus, frontal cortex, and white matter (minimum Bonferroni-corrected *p* value = 2.96 × 10^−4^). Interestingly, we found that despite the weak (but significant) preservation of the *TREM2*-containing module in macrophages, there was a significant overrepresentation of genes related to lytic vacuoles and lysosomes in these data too (minimum Bonferroni-corrected *p* value in human brain = 2.96 × 10^−4^; macrophages = 2.10 × 10^−2^), suggesting that modulation of lysosome activity is a well-preserved *TREM2*-related function though the underlying gene network may be different (Supplementary Fig. 2).

We also found evidence in support of links to the adaptive immune system. There was significant enrichment of genes involved in the regulation of lymphocyte activation in all 10 regional *TREM2*-containing modules (minimum Bonferroni-corrected *p* value = 6.42 × 10^−7^) with evidence for interactions with both B and T cells. The GO term “B cell mediated immunity” was significant in all 10 regional *TREM2*-containing modules (minimum Bonferroni-corrected *p* value = 9.16 × 10^−5^), whereas “T cell activation” was featured in 9 of 10 modules (minimum Bonferroni-corrected *p* value = 8.75 × 10^−5^). Other major microglial functions were also highlighted in this analysis with “antigen processing and presentation via MHC class II” appearing as a highly significant term in all 10 regional *TREM2-*containing modules (minimum Bonferroni-corrected *p* values = 1.15 × 10^−5^) and “the regulation of cytokine production” significant in 8 of 10 modules (minimum Bonferroni-corrected *p* values = 7.68 × 10^−7^). Investigating the *TREM2*-containing modules for the enrichment of GO molecular functions, highlighted the involvement of G-protein-coupled nucleotide receptor signalling in 8 of 10 brain regions (including the hippocampus, Bonferroni corrected *p* value = 3.46 × 10^−4^). Interestingly, genes related to purinergic signaling featured more prominently in the *TREM2*-containing modules than those involved in IgG binding, cytokine signaling, or MHC class II receptor activity.

### 3.8 Insights into TREM2-related signaling pathways

**Fig. 5:**
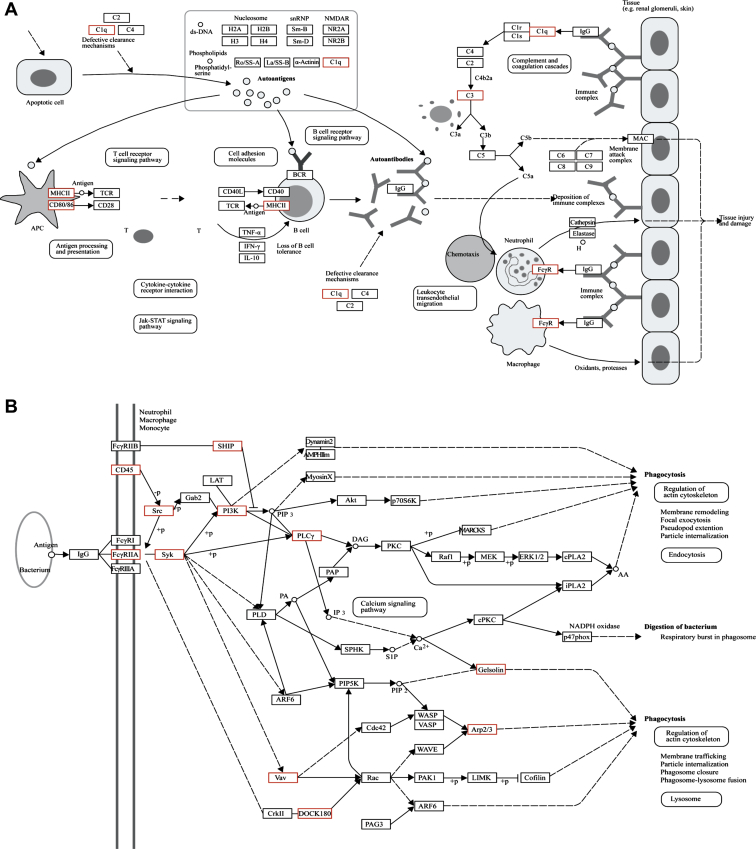
Enrichment of “core” *TREM2*-related genes within Kyoto Encyclopedia of Genes and Genomes (KEGG) pathways. (A) “Fc gamma R-mediated phagocytosis” pathway with proteins/complexes containing “core” *TREM2*-related genes highlighted with a red box (Kanehisa et al., 2012; Kanehisa and Goto, 2000). (B) “Systemic lupus erythematosus” pathway with proteins/complexes containing “core” *TREM2*-related genes highlighted with a red box (Kanehisa et al., 2012; Kanehisa and Goto, 2000).

To obtain insights into *TREM2*-related signaling, we chose to focus on a “core set” of 156 *TREM2*-related genes (defined on the basis of module membership above the 80^th^ quantile in at least 1 brain region). We used this set to look for enrichment of canonical gene sets defined by the Kyoto Encyclopedia of Genes and Genomes (KEGG) and relating to known pathways (Kanehisa and Goto, 2000; Kanehisa et al., 2012). We identified 11 pathways with a Bonferroni-corrected *p* value of <0.05. After accounting for significant overlaps in the gene memberships of these pathways (as calculated within the Molecular Signatures Database v3.1, http://www.broadinstitute.org/gsea/msigdb/index.jsp), we identified 2 independent pathways of interest, the “Fc gamma R-mediated phagocytosis” (Bonferroni-corrected *p* value = 4.72 × 10^−4^) and “Systemic lupus erythematosus” pathways (Bonferroni-corrected *p* value = 3.46 × 10^−4^). These are depicted in Fig. 5A and B with the proteins/complexes containing “core” *TREM2*-related genes highlighted with a red star. Combining these pathways together would explain how *TREM2* located on microglia might act to respond to apoptotic material and immune complexes in their micro-environment through the recognition of the C1Q complex and signal via the ITAM-src-signaling pathway to initiate phagocytosis of the material, but also potentially other processes (including antigen presentation). Because 2 ITAM-containing adaptor proteins are members of the “core” gene set, namely *FCER1G* and *TYROBP*, in theory at least either could associate with TREM2 protein. Similarly, on the basis of *TREM2*-module membership, downstream signaling is most likely to occur through the src kinase, *LYN* (a member of the “core” gene set). However, it is worth noting that *HCK* is a member of the *TREM2*-containing module in medulla and white matter suggesting potential regional heterogeneity in the use of src kinases.

**Fig. 6:**
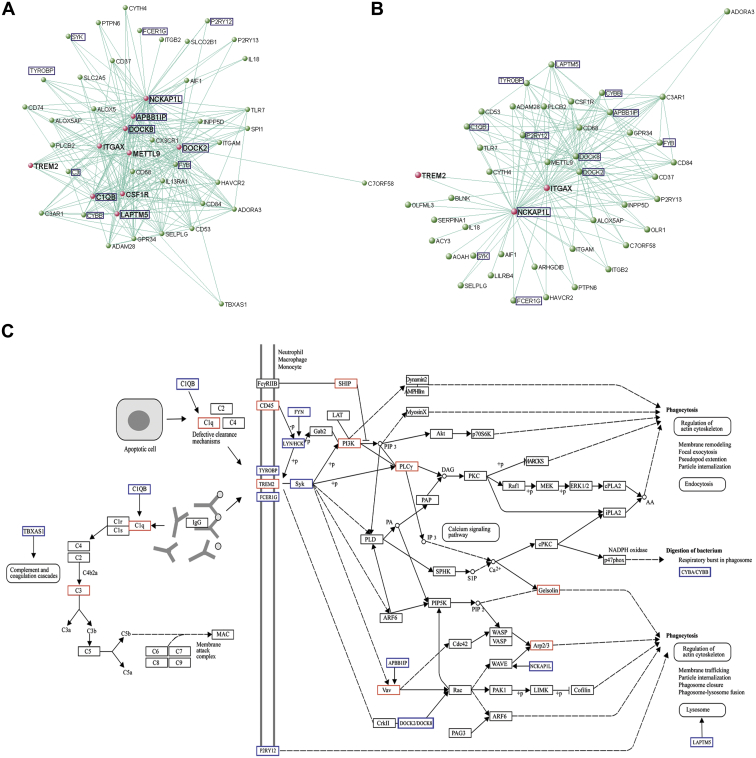
Insights into *TREM2*-related signaling using data-driven network constructions and information provided by Kyoto Encyclopedia of Genes and Genomes (KEGG) pathway analysis. (A) Network depiction of the *TREM2*-containing module in HIPP, showing all genes connected with a topological overlap measure (TOM) >0.10. *TREM2* is highlighted in red, together with all of the genes that are directly connected to it, based on the TOM cut-off used. Genes that are featured in our proposed *TREM2* signaling pathway are boxed in blue. (B) Network depiction of the *TREM2*-containing module in CRBL, showing all genes connected with a TOM >0.10. As above, *TREM2* is highlighted in red, together with the genes that are directly connected to it, based on the TOM cut-off used. Genes that are featured in our proposed *TREM2* signalling pathway are boxed in blue. (C) Proposed *TREM2*-signaling pathway in human brain adapted from the KEGG pathways for “Fc gamma R-mediated phagocytosis” and “Systemic lupus erythematosus.” Genes/complexes highlighted by data-driven network analyses are boxed in blue.

Because the use of canonical pathways is limited by our existing knowledge base, we also chose to visualize gene interconnection within the *TREM2*-containing module using VisANT. Connections among genes was defined on the basis of TOM and genes were “connected” if the TOM was greater than 0.10. Given the involvement of *TREM2* in late-onset AD, we focused on the *TREM2*-containing module in the hippocampus, a region that undergoes severe neurodegeneration, and contrasted this with a relatively unaffected region, the cerebellum (Fig. 6A and B). Based on a TOM of >0.10, we identified 45 genes connected to *TREM2* in hippocampus and similarly 45 genes in cerebellum. As would be expected, both gene lists included members already known to be part of the *TREM2* intracellular signaling pathway (including *TYROBP* and *SYK*) and many genes that were previously identified within the canonical KEGG pathways. Interestingly, both lists also included 2 purinergic receptors, *P2RY12* and *P2RY13*. The *P2YR12* is already known to be expressed on microglia and has been shown to play a critical role in the chemotactic response to ATP released by apoptotic and dying cells (Haynes et al., 2006; Pocock and Kettenmann, 2007). In the case of the hippocampus, this list included 9 genes (*NCKAP1L*, *APBB1IP*, *CSF1R*, *DOCK2*, *DOCK8*, *ITGAX*, *C1QB*, *LAPTM5,* and *METTL9*) with “direct” connection to *TREM2* (as marked in pink and using the cut off of TOM >0.10). Only 2 such genes were identified in cerebellum, *NCKAP1L* and *ITGAX.* Interestingly, 5 of the genes with “direct” connection in hippocampus have been implicated in cytoskeletal rearrangements and cell adhesion. *NCKAP1L* (also known as HEM1) encodes a member of the HEM family of tissue-specific trans-membrane proteins and forms part of the Scar/WAVE complex, which plays an important role in regulating cell shape and has been shown to be essential for actin polymerization and myosin regulation during neutrophil chemotaxis (Weiner et al., 2006). Similarly *DOCK2* is known to be involved in remodeling of the actin cytoskeleton required for lymphocyte migration (Sanui et al., 2003), and the biological functions of *DOCK8* include regulation of cell migration and morphology (Harada et al., 2012; Mizesko et al., 2013). *APBB1IP* (also known as RIAM) encodes the Rap1-GTP-interacting adaptor molecule (*RIAM*) and has been implicated in mediating changes in the actin cytoskeleton after integrin-mediated adhesion acting through the Vav2-RhoA-ROCK-myosin light chain pathway (Hernandez-Varas et al., 2011; Lafuente et al., 2004). Finally, *ITGAX* encodes the integrin alpha X chain protein, which forms part of the alpha X beta 2 complex that has been implicated in the adherence of neutrophils and monocytes to stimulated endothelium cells, and in the phagocytosis of complement-coated particles (Hynes, 1992; Schoenwaelder and Burridge, 1999; van Lookeren Campagne et al., 2007). Although, the remaining 4 genes (*CSF1R*, *LAPTM5*, *C1QB,* and *METTL9*) had no obvious common features, the identification of *CSF1R* is notable, as the TREM2-TYROBP protein complex has recently been implicated in *CSF1R* signaling providing a biological basis for the high connection between *CSF1R* and *TREM2* (Otero et al., 2009). *C1QB* has already been highlighted within the canonical pathway for “Systemic lupus erythrematosus” and *LAPTM5* (lysosomal protein transmembrane 5) is a transmembrane receptor that is associated with lysosomes. Using the information provided by both the investigation of canonical pathways and data-driven network construction, it is possible to build a potential *TREM2*-signaling pathway for human microglia (Fig. 6C) characterized by a central role for *TREM2* in the response to C1Q-containing complexes and ATP/ADP in the micro-environment and the subsequent cytoskeletal changes that are required not only for phagocytosis, but also surveillance and chemotaxis.

### 3.9 Relative enrichment of genes already linked to Alzheimer's disease among genes within the TREM2-containing modules

The identification of variants in *TREM2* as an important cause of late-onset Alzheimer's disease raises the possibility that genes that are highly co-expressed with *TREM2* might also be candidate genes for AD. One way of assessing the validity of this approach is to look at the enrichment of genes already genetically linked to AD among genes within the *TREM2*-containing modules in human brain. Using HUGEnavigator (http://www.hugenavigator.net/HuGENavigator/home.do) (Yu et al., 2008), which uses text mining algorithms to search PubMed and to create an up-to-date knowledge base for exploring genetic associations, we can identify 1499 genes that have been linked to AD. From this list, 1004 genes were studied within our WGCNA analysis of human brain. We looked for enrichment of these AD-related genes within a “core set” of 156 *TREM2*-related genes (defined on the basis of module membership above the 80^th^ quantile in at least 1 brain region). We found a highly significant enrichment of AD-related genes among the “core set” of *TREM2*-connected genes (χ^2^ test with Yates correction *p* value = 3.67 × 10^−10^). Among the genes accounting for this enrichment were *MS4A6A*, which is strongly implicated in AD through recent GWAS (Hollingworth et al., 2011; Naj et al., 2011) and *APBB1IP* (Morgan et al., 2007) (full details provided in Supplementary Table 6). We used a similar approach to look for enrichment of genes implicated in other neurological disorders, namely Parkinson's disease (PD), multiple sclerosis (MS), and motor neuron disease (MND), on the basis that *TREM2*-related microglial activity might be a general contributing factor in the pathogenesis of multiple disorders (Supplementary Table 6). Although there was no evidence for enrichment of PD-related genes (χ^2^ test with Yates correction *p* value = 0.92), there was evidence for enrichment of MS (χ^2^ test with Yates correction *p* value = 7.50 × 10^−15^) MND-related genes (χ^2^ with Yates correction, *p* = 4.85 × 10^−3^). These findings suggest that among the list of “core genes,” there might be as-yet-unidentified AD-related genes, and also (albeit indirectly) validate previously identified AD candidate genes.

## 4 Discussion

The discovery of variants in *TREM2* as a predisposing factor for late-onset AD has opened up a potential new avenue for the understanding of this disease (Guerreiro et al., 2012; Jonsson et al., 2012). To realize that potential an improved appreciation for the “normal” function of *TREM2* in the human brain is required, particularly because increased risk is thought to be mediated through reduced *TREM2* function. To this end, we used a large microarray data set generated from neuropathologically control post-mortem tissue to analyze *TREM2* expression, and we used WGCNA analysis to identify highly co-expressed genes in 10 brain regions, including the hippocampus. This approach, albeit not without its drawbacks, allowed us to obtain insights into *TREM2* function, not in an isolated cell type but in the context of whole human brain tissue. We demonstrate that a highly preserved *TREM2*-containing module of gene expression exists in all brain regions and relates to a microglial expression signature. Importantly, *TREM2* is a hub gene in this module in 5 brain regions, including the hippocampus, suggesting that it is capable of driving module function in these regions at least. Given that the *TREM2*-containing modules were highly enriched for genes related not only to the innate immune system (the major recognized function of *TREM2*), but also the adaptive immune system widens the functional processes related to *TREM2*. Furthermore, close inspection of the genes with the highest connectivity to *TREM2*, provided insights into *TREM2*-related signaling, suggesting that this gene has a central role in mediating changes in the cytoskeletal structure in response to damage-related signals (such as C1Q and ATP/ADP) within the microglial micro-environment. Finally, we demonstrate significant enrichment of candidate genes for AD, MS, and, to a lesser extent, MND within the core genes of the *TREM2*-containing modules, implying, first, that these neurological diseases may share common pathways centred on microglial function and, second, that among the list of genes identified by this study are as-yet-unknown disease-relevant genes.

The basis of this study was microarray data generated from 788 human brain samples originating from 101 neurologically and neuropathologically control individuals collected from a single brain bank, the MRC Sudden Death Brain Bank in Edinburgh, and covering 10 brain regions. These data set allowed us to confidently quantify *TREM2* expression across the human brain, demonstrate significant regional differences, and assess age-related changes in expression. Given that previous studies, not to mention this one, have found that *TREM2* is almost exclusively expressed on microglia in human brain (Colonna, 2003), it seems likely that the regional differences in *TREM2* expression relate to regional differences in either overall microglial density or a subpopulation of microglia. Certainly the higher expression of *TREM2* in white matter versus gray matter samples, and the high *TREM2* expression in hippocampus, mirror known regional differences in microglial density in human brain (Mittelbronn et al., 2001). This interpretation of the *TREM2* expression data would imply that the suggestive age-related increase in *TREM2* expression found in 3 brain regions may also reflect changes in microglial density, again perhaps of a specific sub-population. Although we were unable to find evidence in support of this expression pattern within existing publicly accessible data sets, there is evidence in support of age-related increases in microglia in the normal human brain (Conde and Streit, 2006) and a growing literature to suggest age-related changes in microglial function (Miller and Streit, 2007).

We next used WGCNA to group genes into modules that have strongly co-varying expression patterns, to identify biologically relevant features of the data. This approach allowed us to detect a *TREM2*-containing gene module, which was not only well preserved within all 10 brain regions studied but which could also be replicated in an independent human brain expression data set generated using an unrelated expression platform. In keeping with previous published analyses, this gene module, despite the use of heterogeneous samples (e.g., cerebellar cortex), related to a specific cell type, namely microglia, as demonstrated by the presence of multiple microglial markers within the *TREM2*-containing modules in all human brain samples. We were unable to clearly establish the predominant activation state of the microglia, with the detection of both markers of the M1 activation state (*FCGR2A*, *FCGR2B*, *FCGR3B,* and *CXCL10*) and M2a activation state in a subset of regions (*IGF1*, *PDGFB*, *PDGFC*, *TGFB1,* and *MSR1*). This complexity is likely to arise at least in part from the use of post-mortem brain tissue and the related variability in the mode of death of the individuals sampled. Although all samples originated from individuals undergoing sudden death, some modes of death may be associated with specific forms of microglial activation either due to common preceding events (e.g., angina preceding a myocardial infarction) or more directly. However, it should also be recognized that the understanding of microglial activation states is incomplete (Hanisch and Kettenmann, 2007; Kettenmann et al., 2013), making the identification and use of state-specific markers difficult.

Because studying human microglial function is challenging, we also used publicly available monocyte, dendritic cell, and macrophage microarray data sets to explore *TREM2* expression and assess the validity of these cell types in modeling *TREM2* function in human brain (Barreiro et al., 2012; Fairfax et al., 2012; Smith et al., 2009). In agreement with the existing literature, we detected *TREM2* in dendritic cells and macrophages, but not in monocytes (Colonna, 2003). Although we were able to identify a *TREM2*-containing module in both dendritic cells and macrophages, there was no significant overlap in gene membership among the 2 cell types. There was evidence for weak but significant overlap in the *TREM2*-containing modules in macrophages and brain (as represented by the *TREM2*-containing module in the hippocampus). On deeper inspection, the major common element between these modules was the significant enrichment of lysosome-related genes. These findings suggest that using macrophages and dendritic cells to study *TREM2* function in human brain may not be helpful. Furthermore, these findings highlight the unique properties of microglia, as compared to related myeloid cell types, in keeping with their distinct developmental origin (Chan et al., 2007).

As a result of these findings, we focused our efforts on studying the features of the *TREM2*-containing modules in human brain. We found that *TREM2* was a hub gene in 5 brain regions, namely, the hippocampus, white matter, substantia nigra, and putamen, suggesting that *TREM2* is capable of driving microglial functions in these regions. Although these findings do not fully explain the regional distribution of pathology in either Nasu-Hakola disease or AD, the “hub” role of *TREM2* in white matter and hippocampus is certainly consistent with the severe leukoencephalopathy in the former and the selective loss of hippocampal neurons that characterize the latter. Using GO enrichment analysis to investigate module membership, we confirmed the well-recognized role of *TREM2* in the innate immune response and related that to lysosome activity. However, this analysis also revealed the significant enrichment of genes related to lymphocyte activity (T and B cell), antigen presentation and cytokine release, which may be equally important in human brain.

To obtain further insights into *TREM2*-related signaling, we focused on a “core” group of 156 *TREM2*-related genes and used this gene list to look for enrichment of canonical gene sets defined by KEGG and relating to known pathways. Using this approach we identified 2 independent pathways of interest, the “Fc gamma R-mediated phagocytosis” and “Systemic lupus erythematosus” pathways. Because canonical pathways are limited by our existing knowledge base, we also visualized gene connectivity within the *TREM2*-containing module using VisANT in hippocampus and cerebellum. This approach not only identified genes highlighted within the KEGG pathways, but also identified additional genes of interest based on their high connectivity to *TREM2* particularly in the hippocampus. The predominant common theme among these genes is their involvement in cytoskeletal regulation. For example, *NCKAP1L* (also known as HEM1), a member of the Scar/WAVE complex, plays an important role in regulating cell shape, and is essential in neutrophil chemotaxis (Weiner et al., 2006). Similarly *DOCK2* is known to be involved in remodeling of the actin cytoskeleton required for lymphocyte migration (Sanui et al., 2003) and the biological functions of *DOCK8*, include regulation of cell migration (Harada et al., 2012; Jabara et al., 2012; Lambe et al., 2011). Interestingly, disruptions in this gene have also been linked to mental retardation and developmental disabilities (Griggs et al., 2008). *APBB1IP* encodes the Rap1–GTP-interacting adaptor molecule (*RIAM*) and is involved in mediating changes in the actin cytoskeleton after integrin-mediated adhesion, and is already a candidate gene for late-onset AD based on linkage analyses at chromosome 10 (Hernandez-Varas et al., 2011; Lafuente et al., 2004; Morgan et al., 2007).

This approach also highlighted a range of damage-related signals, namely C1Q complexes, ATP/ADP, and CSF-1, through the identification of *C1QB*, *P2RY12*, *P2RY13,* and *CSF1R*. Although the role of C1Q in microglial activation is well understood, there is emerging evidence for purinergic signaling. Activation of the *P2RY12* is of key importance in mediating the chemotactic response of microglia to ATP/ADP released from apoptotic cells (Haynes et al., 2006; Pocock and Kettenmann, 2007). *CSF1R*, which encodes a cell surface receptor primarily for the cytokine CSF-1, regulates the survival, proliferation, differentiation, and function of mononuclear phagocytic cells, including microglia, and this gene is of particular interest because the TREM2-TYROBP protein complex has recently been implicated in *CSF1R* signaling (Otero et al., 2009). Furthermore, mutations in this gene are a cause of hereditary diffuse leukoencephalopathy with spheroids (HDLS), an autosomal-dominant central nervous system white matter disease with some features in common with Nasu-Hakola disease (Rademakers et al., 2011). Thus, combining these 2 major themes (i.e., damage-related signals and cytoskeletal regulation) would suggest that *TREM2* plays a key role in mediating changes in the microglial cytoskeleton required to initiate not only phagocytosis of apoptotic material but also other processes such as microglial migration to injury or plaque deposition sites (Nimmerjahn et al., 2005).

Finally, but most importantly, we demonstrate a significant enrichment of genes already implicated in AD, MS, and, to a lesser extent, MND (as documented by the HUGEnavigator database (Yu et al., 2008)) among the core genes of the *TREM2*-containing gene modules of human brain. This finding is important for 3 reasons. First, it places a number of known AD risk genes, namely *MS4A6A* and *TREM2,* in the same functional gene module, allowing us to begin to identify important pathophysiological pathways as opposed to single genes. Second, the enrichment of known MS, and to a lesser extent, MND-related genes (but not PD-related genes) suggests that the traditional classification of primary and secondary neurodegenerative diseases may be artificial and that the underlying pathophysiological processes converge (in at least a subset of neurological disorders) and center on microglial function. Third, this analysis implies that among the list of identified genes, there may as yet unidentified risk genes for AD and potentially other neurological disorders. Thus, this study provides important insights into *TREM2*-related signaling, which could be used as a platform for further experiments into the pathophysiological basis of late-onset Alzheimer's disease.

## Disclosure statement

The authors have no conflicting financial, personal, or professional interests.

## Figures and Tables

**Table 1:** Module detection in each brain region: Sample sizes, number of modules, and modules sizes, by brain tissue

Brain region	Samples after outlier removal (n)	Modules (n)	Module size (range)
CRBL	76	23	61–5373
FCTX	83	23	71–5072
HIPP	86	32	46–4193
MEDU	88	17	60–4342
OCTX	77	34	52–3705
PUTM	77	20	38–6729
SNIG	65	13	118–4072
TCTX	72	22	78–2908
THAL	81	19	56–2771
WHMT	83	17	69–4387
Total	**788**		

Brain regions are available regions for human brains. Key: CRBL, cerebellum; FCTX, frontal cortex; HIPP, hippocampus; MEDU, medulla (specifically inferior olivary nucleus); OCTX, occipital cortex (specifically primary visual cortex); PUTM, putamen; SNIG, substantia nigra; TCTX, temporal cortex; THAL, thalamus; WHMT, intralobular white matter.

**Table 2:** Number of genes in the *TREM2*-containing module for each tissue and overlap with *TREM2*-containing modules in other tissues

Tissue	Genes in module (n)	CRBL	FCTX	HIPP	MEDU	OCTX	PUTM	SNIG	TCTX	THAL	WHMT
CRBL	226	—	132 (58.4%)	145 (64.2%)	208 (92%)	147 (65%)	113 (50%)	196 (86.7%)	151 (66.8%)	153 (67.7%)	218 (96.5%)
FCTX	151	132 (87.4%)	—	117 (77.5%)	144 (95.4%)	127 (84.1%)	100 (66.2%)	143 (94.7%)	122 (80.8%)	125 (82.8%)	149 (98.7%)
HIPP	175	145 (82.9%)	117 (66.9%)	—	162 (92.6%)	136 (77.7%)	101 (57.7%)	158 (90.3%)	134 (76.6%)	128 (73.1%)	169 (96.6%)
MEDU	560	208 (37.1%)	144 (25.7%)	162 (28.9%)	—	176 (31.4%)	122 (21.8%)	283 (50.5%)	196 (35%)	176 (31.4%)	426 (76.1%)
OCTX	186	147 (79%)	127 (68.3%)	136 (73.1%)	176 (94.6%)	—	106 (57%)	162 (87.1%)	139 (74.7%)	135 (72.6%)	176 (94.6%)
PUTM	133	113 (85%)	100 (75.2%)	101 (75.9%)	122 (91.7%)	106 (79.7%)	—	119 (89.5%)	106 (79.7%)	104 (78.2%)	125 (94%)
SNIG	377	196 (52%)	143 (37.9%)	158 (41.9%)	283 (75.1%)	162 (43%)	119 (31.6%)	—	173 (45.9%)	165 (43.8%)	294 (78%)
TCTX	257	151 (58.8%)	122 (47.5%)	134 (52.1%)	196 (76.3%)	139 (54.1%)	106 (41.2%)	173 (67.3%)	—	134 (52.1%)	194 (75.5%)
THAL	187	153 (81.8%)	125 (66.8%)	128 (68.4%)	176 (94.1%)	135 (72.2%)	104 (55.6%)	165 (88.2%)	134 (71.7%)	—	177 (94.7%)
WHMT	728	218 (29.9%)	149 (20.5%)	169 (23.2%)	426 (58.5%)	176 (24.2%)	125 (17.2%)	294 (40.4%)	194 (26.6%)	177 (24.3%)	—

Percentages are quoted as proportion of the number of genes in the *TREM2*-containing module in the tissue indicated in the rows. Overlaps were tested using Fisher's exact test at a Bonferroni-adjusted value of *p* < 1.1 × 10^−3^ = 0.05/45. All overlaps were found to be highly significant. Key: CRBL, cerebellum; FCTX, frontal cortex; HIPP, hippocampus; MEDU, medulla (specifically inferior olivary nucleus); OCTX, occipital cortex (specifically primary visual cortex); PUTM, putamen; SNIG, substantia nigra; TCTX, temporal cortex; THAL, thalamus; WHMT, intralobular white matter.

**Table 3:** Summary of the module membership (MM) within the *TREM2*-containing modules for each brain tissue for *TREM2* and all transcripts assigned to *TREM2*-containing modules

Data set	Genes in module (n)	*TREM2*		Summary statistics of the MM of the transcripts within the *TREM2*-containing module					
		MM	1–p^th^ quantile	Min	First quartile	Median	Mean	Third quartile	Max
UKBEC									
CRBL	226	0.82	0.17	0.29	0.55	0.68	0.67	0.78	0.95
FCTX	151	0.78	0.26	0.32	0.64	0.72	0.71	0.78	0.94
HIPP	175	0.89	**0.09**	0.20	0.68	0.76	0.74	0.83	0.95
MEDU	560	0.92	**0.03**	0.17	0.51	0.68	0.65	0.8	0.95
OCTX	186	0.76	0.25	0.25	0.55	0.66	0.65	0.76	0.92
PUTM	133	0.88	**0.05**	0.4	0.64	0.73	0.71	0.79	0.92
SNIG	377	0.88	**0.07**	0.31	0.55	0.67	0.67	0.8	0.94
TCTX	257	0.79	0.15	0.22	0.57	0.66	0.66	0.75	0.94
THAL	187	0.86	0.12	0.41	0.63	0.74	0.71	0.81	0.94
WHMT	728	0.9	**0.03**	0.03	0.43	0.6	0.59	0.75	0.95
Colantuoni et al.	315	0.8	0.12	0.20	0.51	0.62	0.62	0.74	0.92

1–p^th^ quantile = rank_MM_(*TREM2*)/module size. The p^th^ quantiles >90^th^ quantile are highlighted in boldface type. For these tissues, *TREM2* shows a major role within the module (hub gene). Key: CRBL, cerebellum; FCTX, frontal cortex; HIPP, hippocampus; MEDU, medulla (specifically inferior olivary nucleus); OCTX, occipital cortex (specifically primary visual cortex); PUTM, putamen; SNIG, substantia nigra; TCTX, temporal cortex; THAL, thalamus; UKBEC, UK Human Brain Expression Consortium; WHMT, intralobular white matter.

**Table 4:** Summary of the results of GO enrichment analysis performed on the *TREM2*-containing modules in each brain region

Key: CRBL, cerebellum; FCTX, frontal cortex; HIPP, hippocampus; MEDU, medulla (specifically inferior olivary nucleus); OCTX, occipital cortex (specifically primary visual cortex); PUTM, putamen; SNIG, substantia nigra; TCTX, temporal cortex; THAL, thalamus; WHMT, intralobular white matter.

## Definitions

### Adjacency matrix

The adjacency matrix defines the co-expression network. It is constructed here from the pairwise Pearson correlations between all genes, raising these correlations to a power beta while maintaining the information on the correlation signs between genes, thus constructing a signed weighted co-coexpression network.

### Connectivity

For each gene, the connectivity (also known as degree) is defined as the sum of its adjacencies with the other network genes. In co-expression networks, the connectivity measures how correlated a gene is with all other network genes.

### Topological overlap matrix (TOM)

Topological overlap measure (TOM) is a pairwise measure of node similarity, that measures how close the neighbors of a gene are to the neighbors of the other gene. Unlike correlation or adjacency measures, which consider each pair of genes in isolation, TOM considers each pair of genes in relation to all other genes in the network, and it reflects the relative interconnectedness of 2 nodes within their local neighbourhood.

### Module eigengene (ME)

The module eigengene is defined as the first principal component of the expression profiles of all genes in a given module.

### Module membership (MM)

For each gene, the module membership (also known as eigengene-based connectivity) is calculated as the correlation of the gene expression profile with the module eigengene (ME) of a given module. If MM of a gene for a given module is close to 0, it means that the gene is not part of that module. On the other hand, if MM is close to 1 or 1, it is highly connected to the module genes. The sign of module membership encodes whether the gene has a positive or a negative relationship with the module eigengene. Highly connected intramodular hub genes tend to have high module membership values to the respective module.
