# Supplementary material for: Insights into TREM2 biology by network analysis of human brain gene expression data
Source: Neurobiol Aging. 2013 Dec;34(12):2699–714. doi: 10.1016/j.neurobiolaging.2013.05.001 (PMC3988951; doi:10.1016/j.neurobiolaging.2013.05.001)
Supplement: Supplementary Data [file mmc1.doc]

**Supplementary Data**

**Supplementary Figure 1**: Age-related effects on *TREM2* expression as demonstrated by Kang et al. (2011).

**Supplementary Figure 2**: Network depiction of the *TREM2-*containing module in macrophages.

**Supplementary Table 1**: Demographic details related to brain donors (submitted as excel spreadsheet)

**Supplementary Table 2**: List of CEL files used (submitted as excel spreadsheet)

**Supplementary Table 3**: Summary of network connectivity in human brain tissues and *TREM2* connectivity

**Supplementary Table 4**: Markers of microglial state analysed (submitted as excel spreadsheet)

**Supplementary Table 5**: Summary of enriched GO terms in the *TREM2*-containing modules in brain (submitted as excel spreadsheet)

**Supplementary Table 6**: Core genes and information regarding their status as disease-related genes (submitted as excel spreadsheet)

**Supplementary Figure 1**: Age-related effects on *TREM2* expression as demonstrated by Kang et al. (2011).

Graph to show TREM2 mRNA expression levels in six brain regions during the course of human brain development and during adult life (period 13 – 15). The expression levels are based on exon array experiments and are plotted on a log2 scale (Kang et al., 2011). The brain regions analyzed are the striatum (STR), amygdala (AMY), neocortex (NCX), hippocampus (HIP), mediodorsal nucleus of the thalamus (MD), and cerebellar cortex (CBC).

**Supplementary Figure 2**: Network depiction of the *TREM2-*containing module in macrophages.

**
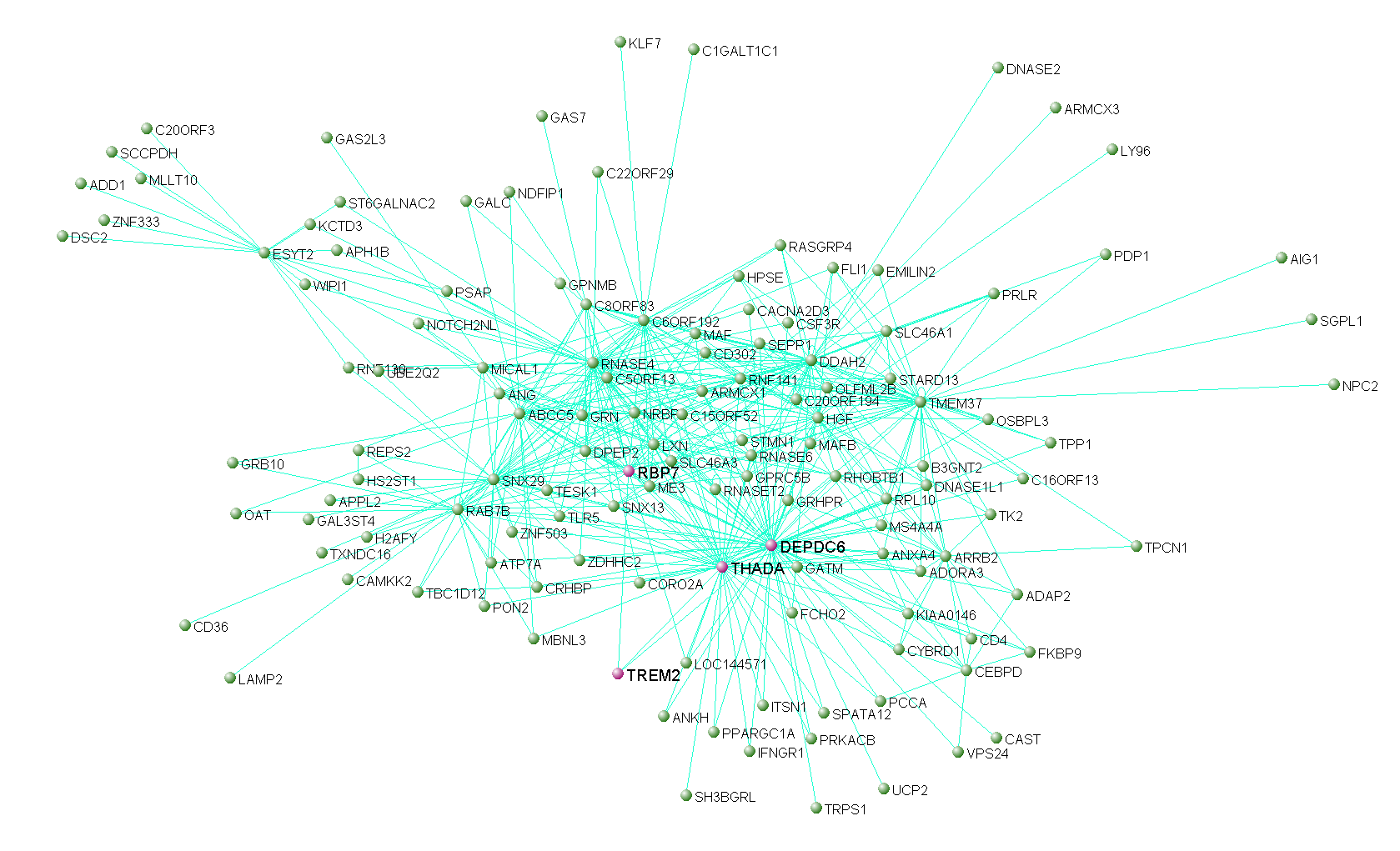
**

All genes connected with a TOM > 0.10 are depicted. *TREM2* is highlighted in red together with all the genes that are directly connected to it, based on the TOM cut-off used.

**Supplementary Table 3: Summary of network connectivity in human brain tissues and *TREM2* connectivity**

| **Brain tissue** | **Min** | **1st**  **quartile** | **Median** | **Mean** | **3rd**  **quartile** | **Max** | ***TREM2***  **connectivity** |
| --- | --- | --- | --- | --- | --- | --- | --- |
| CRBL | 5.5 | 14.3 | 21.0 | 34.8 | 37.7 | 271.6 | 34.2 |
| FCTX | 5.4 | 18.2 | 30.1 | 47.4 | 56.9 | 330.1 | 24.5 |
| HIPP | 5.3 | 21.8 | 36.8 | 46.9 | 59.8 | 271.3 | **81.4** |
| MEDU | 5.2 | 23.9 | 47.3 | 64.8 | 87.8 | 339.8 | **105.9** |
| OCTX | 5.9 | 19.3 | 29.2 | 38.0 | 45.9 | 220.8 | 20.2 |
| PUTM | 5.5 | 19.7 | 37.9 | 67.9 | 88.6 | 469.8 | 37.8 |
| SNIG | 6.4 | 24.9 | 47.0 | 63.3 | 86.0 | 327.6 | 74.4 |
| TCTX | 5.3 | 19.0 | 31.5 | 44.6 | 60.0 | 240.7 | 45.8 |
| THAL | 5.3 | 24.8 | 50.5 | 69.6 | 99.2 | 324.6 | 60.0 |
| WHMT | 5.3 | 19.8 | 34.6 | 44.9 | 58.7 | 276.9 | **84.7** |
